# Promoter Hypermethylation Is Associated with Reduced Nrf2 and Antioxidant Enzyme Expression in Mandibular Condylar Cartilage in Mice

**DOI:** 10.3390/antiox15070854

**Published:** 2026-07-06

**Authors:** Hisano Ujiie, Hiroyuki Kanzaki, Mao Katayama, Tomomi Ida, Syunnosuke Tohyama, Miho Shimoyama, Yuta Katsumata, Chihiro Arai, Misao Ishikawa, Hiroshi Tomonari

**Affiliations:** 1 Department of Orthodontics, School of Dental Medicine, Tsurumi University, Yokohama 230-8501, Japan; 2 Department of Orthodontics, Tohoku University Hospital, 4-1 Seiryo-cho, Aoba-ku, Sendai 980-8575, Japan; 3 Department of Anatomy, School of Dental Medicine, Tsurumi University, Yokohama 230-8501, Japan

**Keywords:** cartilage, antioxidant, Nrf2, epigenome, reactive oxygen species (ROS), promoter, DNA methylation, temporomandibular joint, mechanical stress, osteoarthritis

## Abstract

Mandibular condylar cartilage (MCC) exhibits greater susceptibility to mechanical stress-induced degeneration than tibial articular cartilage (TAC). This study investigated whether differential epigenetic regulation of nuclear factor erythroid 2-related factor 2 (Nrf2), a master regulator of antioxidant responses, is associated with distinct antioxidant capacities between these cartilage types. Cartilage tissues from 5-week-old male ICR mice (*n* = 16 for gene analyses, n = 8 for protein analyses) were obtained using laser microdissection. Gene and protein expression was analyzed by microarray, real-time RT-PCR, and immunohistochemistry. DNA methylation of the Nrf2 promoter was evaluated using pyrosequencing and high-resolution melting analysis. Nrf2 expression in MCC was approximately 1/10 that in TAC at mRNA level and only 5% at protein level. Downstream antioxidant enzymes (NQO1, G6PD, HO-1) showed significantly reduced expression in MCC. Oxidative DNA damage marker 8-OHdG was significantly elevated in MCC compared to TAC (20.0% vs. 10.7%, *p* < 0.05). The Nrf2 promoter region showed higher DNA methylation levels in MCC, confirmed by high-resolution melting analysis. Higher Nrf2 promoter methylation in MCC is associated with reduced antioxidant capacity and elevated oxidative damage. This epigenetic–antioxidant relationship may contribute to MCC’s vulnerability to mechanical stress-induced degeneration and represents a potential therapeutic target for temporomandibular joint disorders.

## 1. Introduction

Cartilage tissue is an important component that allows for smooth movement of joints and is composed of chondrocytes and their extracellular matrix [1]. Mandibular condylar cartilage (MCC) and tibial articular cartilage (TAC) differ in several respects. MCC is derived from neural crest cells, whereas TAC originates from mesoderm [2]. MCC is enriched in type II collagen and proteoglycans, while TAC contains more type I collagen [3]. Importantly, reactivity to mechanical stress differs between these cartilage types. MCC growth is inhibited by long-term compressive force loading [4,5], whereas TAC maintains resistance to compressive forces without growth inhibition [6]. Despite MCC’s higher type II collagen content and greater chondrocyte proliferative capacity [7], the molecular mechanisms underlying this differential mechanical stress tolerance remain unclear.

Cartilage tissue is particularly vulnerable to oxidative stress due to its avascular nature and hypoxic environment [8]. Oxidative stress is a major factor in cartilage degeneration and osteoarthritis pathogenesis [9]. Reactive oxygen species (ROS) exert cytotoxic effects through lipid peroxidation and oxidative damage to proteins and DNA [10,11]. To counter these stressors, cells possess defense mechanisms that induce cytoprotective enzymes and ROS scavenging [12,13].

Nuclear factor erythroid 2-related factor 2 (Nrf2) is a transcription factor that plays a central role in the oxidative stress response by regulating antioxidant enzyme expression. Recent studies have demonstrated that Nrf2 signaling is crucial for maintaining cartilage homeostasis. Inhibition of Nrf2 signaling results in loss of temporomandibular joint osteoarthritis protection [14], while Nrf2 activation mitigates mandibular condylar cartilage destruction [15]. These findings suggest that Nrf2 and its antioxidant axis play an important role in maintaining MCC homeostasis.

Nrf2 expression is regulated by epigenetic mechanisms, with DNA methylation of the Nrf2 promoter region suppressing its expression in various cell types [16,17]. Epigenetic regulation through DNA methylation and histone modifications plays a crucial role in cartilage homeostasis, affecting chondrocyte differentiation and matrix metabolism [18,19]. However, whether tissue-specific differences in Nrf2 promoter methylation contribute to the distinct mechanical stress tolerance between MCC and TAC remains unclear.

We hypothesized that Nrf2 expression in MCC and TAC is differentially epigenetically regulated, resulting in lower antioxidant capacity in MCC compared to TAC, and that this difference contributes to the distinct mechanical stress tolerance between these cartilage types. The objective of this study was to investigate the epigenetic regulation of Nrf2 and antioxidant enzyme expression in MCC and TAC, and to elucidate their relationship to differential mechanical stress tolerance.

## 2. Materials and Methods

### 2.1. Reagents

All reagents were purchased from Fujifilm Wako Chemicals (Tokyo, Japan), unless otherwise stated.

### 2.2. Experimental Animals

Five-week-old male ICR mice (approximately 25 g body weight, n = 24, CLEA Japan, Tokyo, Japan) were used for this study. All animal experimental procedures were approved by the Animal Research Committee of Tsurumi University (approval No. 27P070, approved on 14 July 2016, and approval No. 20A010, approved on 17 April 2020). This study was performed in accordance with relevant guidelines and regulations. All methods are reported in accordance with ARRIVE guidelines.

### 2.3. Preparation of Tissue Sections

Mice were sacrificed by cervical dislocation. For gene analyses, mandible and tibia of 16 mice were immediately excised, and the specimens were embedded in OCT compound (Sakura Fineteck Japan, Tokyo, Japan) and rapidly frozen in isopentane cooled by liquid nitrogen. Undecalcified nonfixed serial frozen sections (5 μm thick) of MCC and TAC were prepared using a super-hard tungsten steel knife (Meiwa Shoji Ltd., Tokyo, Japan) in −25 °C cryostat (Leica Microsystems, Wetzlar, Germany). The sections were collected individually on a 1.35 μm thick polyethylene naphthalene membrane with an adhesive (LMD film II, Leica Microsystems) and used for laser microdissection (LMD).

For protein expression analyses, mandible and tibia of the remaining 8 mice were decalcified with 10% ethylene diamine-tetra acetic acid in PBS for 3 weeks at 4 °C, dehydrated, and embedded in paraffin. Serial sections (6 μm thick) of MCC and TAC were then prepared and used for immunohistochemical staining.

### 2.4. Laser Microdissection (LMD)

Cartilage tissues of MCC and TAC were microdissected and collected using laser microdissection (P.A.L.M. Microlaser Technologies, Bernried, Germany). Microdissected cartilage tissues (5 sections, approximately 15,000 μm^2^ each) were pooled and used for genomic DNA and RNA extraction. The boundaries of the MCC and TAC regions were photographed after laser microdissection (Supplementary Figure S1A,B).

### 2.5. Extraction of Genomic DNA and RNA

Genomic DNA and RNA were extracted using the QIAamp Fast DNA Tissue Kit (Qiagen, Germantown, MD, USA) and RNeasy plus Micro kit (Qiagen) following the manufacturers’ instructions, respectively. Quality control of the microdissected cartilage and the extracted nucleic acids is shown in Supplementary Figure S1. Genomic DNA purity was evaluated by spectrophotometric A260/280 absorbance ratio (NanoDrop, Thermo Inc., Vista, CA, USA; 1.84), and RNA integrity was evaluated by microcapillary electrophoresis using an Agilent Bioanalyzer (Agilent Technologies, Santa Clara, CA, USA); representative electropherograms showing clearly resolved 18S and 28S ribosomal RNA peaks, with minimal evidence of degradation, are shown in Supplementary Figure S1C,D.

Genomic DNA was extracted from a total of n = 8 animals, which were divided into two independent groups of n = 4 animals each; within each group, genomic DNA from MCC and from TAC was pooled separately, yielding one pooled MCC sample and one pooled TAC sample per group. One group’s pooled samples were used for pyrosequencing analysis, and the other group’s pooled samples were used for high-resolution melting (HRM) analysis, both targeting the promoter region of Nrf2. RNA was extracted from a separate total of n = 8 animals (distinct from those used for genomic DNA extraction) and similarly divided into two independent groups of n = 4 animals each, with RNA from MCC and from TAC pooled separately within each group; one group’s pooled RNA samples were used for gene expression analysis by microarray, and the other group’s pooled RNA samples were used for real-time RT-PCR.

### 2.6. Gene Expression Analysis Using Microarray

The details of microarray analysis were described elsewhere [20]. Briefly, RNA samples (n = 4 animals) of MCC and TAC were analyzed using Whole Mouse Genome Microarray 4×44K (Agilent Technologies, Santa Clara, CA, USA) according to the manufacturer’s instructions. Relative gene expressions in MCC against that in TAC were calculated.

### 2.7. Reverse Transcription (RT) and Real-Time RT-PCR Analysis

**Figure 2:**
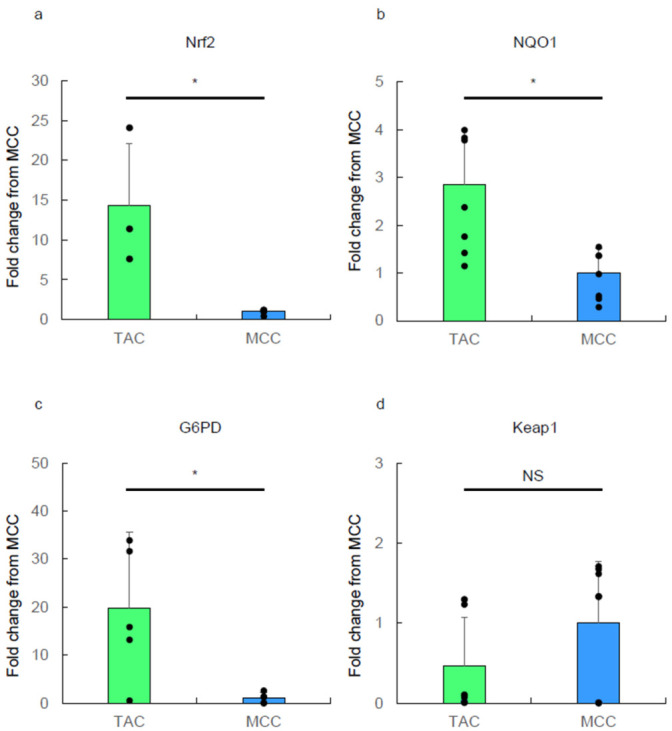
Real-time RT-PCR analysis for antioxidant enzyme expression. Gene expressions of Nrf2 (**a**), NQO1 (**b**), G6PD (**c**), and Keap1 (**d**) were examined using RNA pooled from n = 4 animals per tissue type. Gene expression was normalized relative to Rps18, and fold changes from MCC were calculated. Representative results from one of three independent experiments are shown as the mean ± SD and scatter plots with the full data set of technical replicates (n = 3 for Nrf2, n = 6 for NQO1, n = 5 for G6PD, and n = 9 for Keap1). * *p* < 0.05 between the samples. NS: no statistically significant difference.

RNA was pooled from n = 4 animals per tissue type prior to reverse transcription. Extracted RNA was reverse-transcribed with iScript Reverse Transcription Supermix (Bio-Rad Laboratories, Hercules, CA, USA), and the resulting cDNA was used for gene expression analysis with SsoFast EvaGreen Supermix (Bio-Rad), performed in technical replicate (see Figure 2 legend for the number of replicates per gene). The following primer sets used in this study were from PrimerBank, and primer sequences are as follows: Rps18 (gene ID: 20084): (F) 5′-AGTTCCAGCACATTTTGCGAG-3′ and (R) 5′-TCATCCTCCGTGAGTTTCTCCA-3′; Nrf2 (gene ID: 18024): (F) 5′-GCCCACATTCCCAAACAAGAT-3′ and (R) 5′-CCAGAGAGCTATTGAGGGACTG-3′; NQO1 (gene ID: 18104): (F) 5′-AGGATGGGAGGTACTCGAATC-3′ and (R) 5′-AGGCGTCCTTCCTTATATGCTA-3′; G6PD (gene ID: 14380): (F) 5′-AGGTGACCCTAAGCCGGAC-3′ and (R) 5′-AGGTTTCTTTGGGTAGAAGACCA-3′; Keap1 (gene ID: 50868): (F) 5′-TGCCCCTGTGGTCAAAGTG-3′ and (R) 5′-GGTTCGGTTACCGTCCTGC-3′. Fold changes in gene expression were calculated using the ΔΔCt method with ribosomal protein S18 as a reference.

### 2.8. Immunohistochemical Staining

Paraffin-embedded histological sections were deparaffinized, their endogenous peroxidase activity was blocked by 0.3% hydrogen peroxide in PBS, they were preincubated in BlockAce (Bio-Rad), and they were subsequently incubated with primary antibody. After being rinsed, the sections were incubated with HRP-conjugated secondary antibody (Fortis Life Sciences LLC, Boston, MA, USA). The primary antibodies used in this experiment were as follows: anti Nrf2 antibody (Santa Cruz Biotechnology, Inc., Dallas, TX, USA), anti HO-1 antibody (StressMarq Biosciences Inc., Vancouver, BC, Canada), and anti 8-hydroxydeoxyguanosine (8-OHdG) antibody (Bioss Inc., Woburn, MA, USA). 8-OHdG is a well-established biomarker that accurately reflects endogenous oxidative stress levels and DNA damage in biological systems [21].

To visualize immunoreactivity, the sections were flooded with a diaminobenzidine solution (Vector Laboratories, Newark, CA, USA) under the same reaction conditions among sections. The sections were then observed with a microscope (BZ-9000; Keyence Co., Osaka, Japan). Images were obtained from the n = 8 animals in the protein expression analysis cohort, prioritizing animals from which sections of sufficient staining quality were available for quantification; for some animals, more than one section was quantified. To quantify the immunoreactivity among the sections, 4 to 13 images were analyzed with ImageJ Version 1.54g (plugins: Color Deconvolution; vectors: Haematoxylin and DAB (H DAB); National Institutes of Health, Bethesda, MD, USA).

### 2.9. Pyrosequencing Analysis

**Figure 1:**
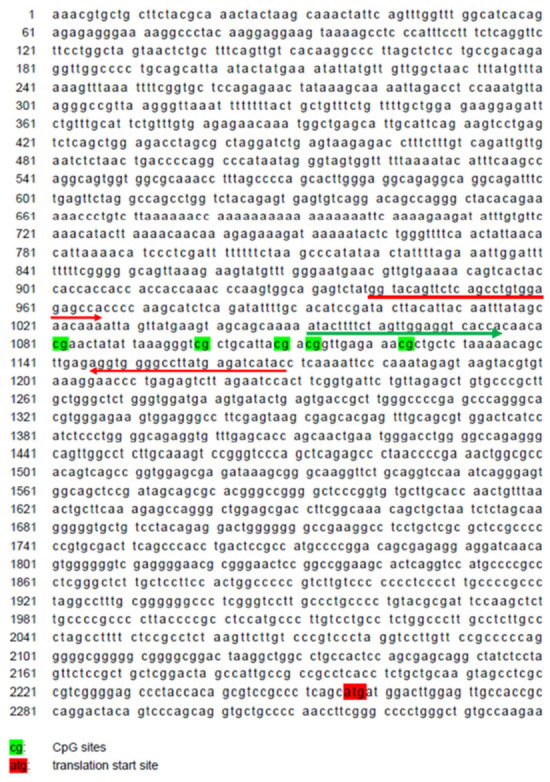
CpG sites in Nrf2 promoter region. The Nrf2 promoter region has 5 CpG sites as marked by green. The red arrow under the sequence indicates PCR primers to amplify the sequence including CpG sites. The green arrow under the sequence indicates the sequencing primer for pyrosequencing. ATG marked by red indicates the translation start site.

Pyrosequencing of the CpG sites in the promoter region of Nrf2 was performed using genomic DNA samples of both MCC and TAC. For each tissue type, genomic DNA extracted from n = 4 animals was pooled prior to bisulfite treatment, yielding one pooled template per tissue type. Firstly, the pooled samples were treated with bisulfite using the EZ DNA Methylation-Lightning kit (Zymo Research, Irvine, CA, USA). Five CpG sites included in the Nrf2 promoter region (Figure 1) [17,22] were PCR-amplified with a primer set (forward: GGTATAGTTTTTAGTTTGTGGAGAGTTA; reverse: ATATAATCTCATAAAACCCCACCT), and were subjected to pyrosequencing on PyroMark ID (QIAGEN), with the sequencing primer (ATATTTTTTTAGTTGGAGGTTA). The percentage methylation at each of the five CpG sites was then calculated for MCC and TAC pooled samples.

### 2.10. High-Resolution Melting (HRM) Analysis

Bisulfite conversion was performed with EpiSight Bisulfite Conversion Kit Ver.2 according to the manufacturer’s instructions. Methylation-Sensitive High-Resolution Melting analysis was performed with SsoFast EvaGreen Supermix (BioRad) and a specific primer set (forward: ATATTTTTTTAGTTGGAGGTTA; reverse: ATATAATCTCATAAAACCCCACCT) using CFX Connect™ Real Time System (Bio-Rad) and Precision Melt Analysis Software Version 1.3 (BioRad). To calculate the percentage of methylation, we constructed and used the following sequence for positive and negative control of methylation: (original: aaaatatttttttagttggaggttattataatacgaattatattaaagggtcgttgtattacgacggttgagaaacgttgttttaaaaatagtttgagaggtggggttttatgagattatatttt; converted: aaaatatttttttagttggaggttattataataTgaattatattaaagggtTgttgtattaTgaTggttgagaaaTgttgttttaaaaatagtttgagaggtggggttttatgagattatatttt). For quantification of methylation levels, a calibration curve correlating Relative Fluorescence Units (RFUs) to methylation percentage was established using standard samples with known methylation levels ranging from 0% to 100%. The methylation percentages of TAC and MCC samples were subsequently determined by interpolating their measured RFU values against this established calibration curve.

### 2.11. Statistical Analysis

All data are presented as the mean and standard deviation (SD). Welch’s *t*-test (two-sample *t*-test assuming unequal variances, Microsoft Excel in Microsoft 365, version 2606, Redmond, WA, USA) was used to compare MCC and TAC for all gene and protein expression analyses. For pyrosequencing analysis, comparisons between MCC and TAC groups were performed using paired *t*-tests. *p* < 0.05 was considered to be statistically significant.

To investigate the differences in CpG methylation levels between MCC and TAC samples across multiple positions, we employed the sign test, a non-parametric statistical method. This test was chosen due to its ability to analyze paired data without requiring assumptions about the underlying distribution of the data. The sign test specifically examines the consistency of directional differences between paired observations, making it suitable for our analysis where we aimed to determine whether one sample consistently exhibited higher methylation levels than the other across all measured positions. For each CpG site (site 1–5), the difference between MCC and TAC methylation percentages was calculated, and the sign of this difference (positive, negative, or zero) was recorded. The null hypothesis stated that there was no consistent difference between samples, with positive and negative differences being equally likely to occur by chance (probability = 0.5). The *p*-value was calculated using the binomial probability distribution based on the number of consistent sign differences observed. Statistical significance was set at *p* < 0.05.

## 3. Results

### 3.1. Microarray Analysis for Nrf2 and Antioxidant Enzymes

Microarray analysis revealed the difference in the expression of some antioxidant enzyme genes between MCC and TAC (Table 1). The expression of Nrf2, the master regulator of antioxidant enzymes, in MCC was almost 1/10 that of TAC. Interestingly, Keap1 expression in both tissues was almost the same and the Nrf2/Keap1 ratio in MCC and TAC was 0.336, and 3.588, respectively. Similarly, the expression levels of almost all examined antioxidant enzymes were quite low in MCC as compared to TAC. These results suggest that antioxidant capacity is lower in MCC as compared to that in TAC.

### 3.2. Gene Expressions of Nrf2 and Antioxidant Enzymes Were Weak in MCC

Gene expression analysis using real-time RT-PCR clearly demonstrated that Nrf2, NQO1, and G6PD expression in MCC was weak as compared to that in TAC (Figure 2a–c). On the other hand, there was no statistically significant difference in Keap1 expression between MCC and TAC (Figure 2d).

### 3.3. Protein-Level Expressions of Nrf2 and Antioxidant Enzymes Were Weak in MCC

**Figure 3:**
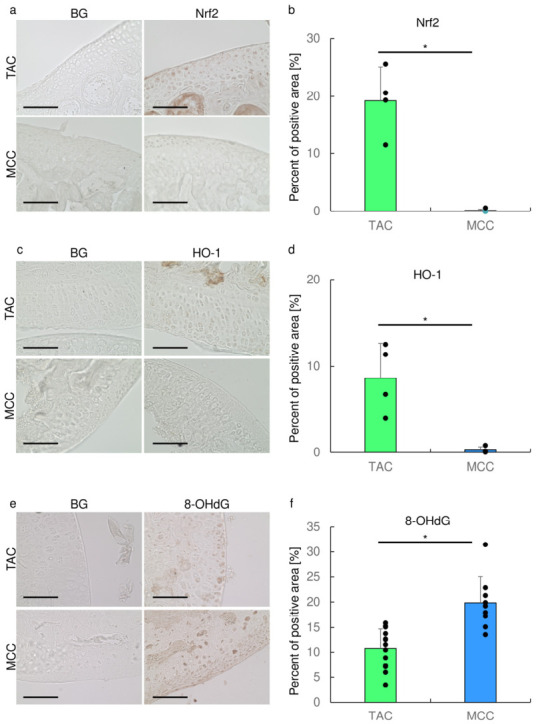
Immunohistochemical staining for Nrf2, HO-1, and 8-OHdG. Representative photographs of immunohistochemical staining for Nrf2 (**a**), HO-1 (**c**), and 8-OHdG (**e**) are shown. BG indicates background-level staining without HRP-conjugated secondary antibody. The upper two panels indicate the sections of TAC, and the lower two panels indicate the sections of MCC. Bar: 100 μm. A quantified percentage of stained area was compared between the samples of TAC and MCC (**b**,**d**,**f**). Results are shown as the mean ± SD and scatter plots with the full data set. * *p* < 0.05 between the samples. Calculations were performed from four images each of TAC and MCC for Nrf2 and HO-1 expression analysis, and from 13 images of TAC and 9 images of MCC for 8-OHdG expression analysis; image numbers reflect the availability of sections of sufficient quality across the n = 8 animals in this cohort, with more than one section quantified for some animals (see Section 2).

We examined protein-level expressions of Nrf2, antioxidant enzyme HO-1, and oxidative stress marker 8-OHdG in MCC and TAC (Figure 3). Consistent with the gene expression analysis, protein-level expression of Nrf2 was significantly reduced in MCC compared to TAC (Figure 3a). The quantification demonstrated that Nrf2 expression in MCC was approximately 5% of that observed in TAC (*p* < 0.05) (Figure 3b). Similarly, antioxidant enzyme HO-1 expression was significantly lower in MCC relative to TAC (Figure 3c,d), with almost undetectable levels in MCC tissues (*p* < 0.05). Subsequently, we investigated 8-hydroxydeoxyguanosine (8-OHdG), a well-established biomarker that accurately reflects endogenous oxidative stress levels and DNA damage in biological systems [21]. 8-OHdG immunoreactivity was observed throughout all layers of MCC, with particularly intense signals in the superficial layers (Figure 3e). Quantitative analysis revealed that 8-OHdG-positive areas were significantly more abundant in MCC compared to TAC (20.0% vs. 10.7%, *p* < 0.05) (Figure 3f). These results suggest that decreased Nrf2 and HO-1 expression in MCC may contribute to elevated oxidative DNA damage, as evidenced by increased 8-OHdG immunoreactivity.

### 3.4. Assessment of Nrf2 Promoter CpG Methylation via Pyrosequencing Analysis

Pyrosequencing analysis revealed the methylation status at five CpG sites in both MCC and TAC samples (Table 2). The MCC sample consistently showed higher methylation percentages compared to the TAC sample. The paired *t*-test revealed a statistically significant difference between MCC and TAC groups (mean difference = 7.64, t(4) = 3.68, *p* = 0.021, Cohen’s dz = 1.65).

The consistency of directional differences between MCC and TAC samples was evaluated using the sign test. For each of the five CpG sites, the sign of the difference (MCC-TAC) was recorded. All five positions showed positive differences, indicating consistently higher methylation in MCC compared to TAC (5/5 positive signs, one-sided *p* = 0.031).

### 3.5. Evaluation of CpG Methylation Status in the Nrf2 Promoter Region by HRM Analysis

**Figure 4:**
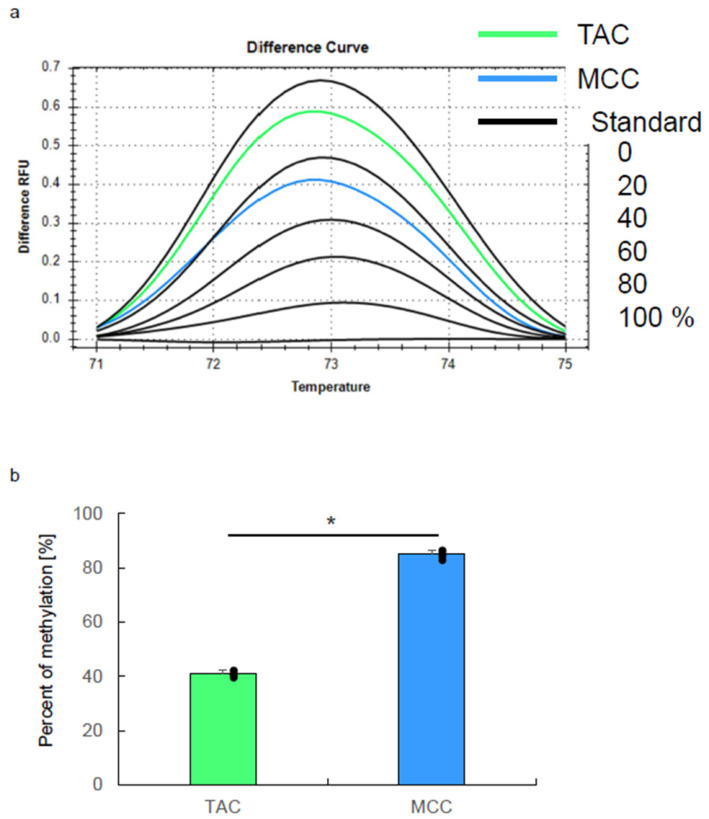
HRM analysis of Nrf2 promoter methylation. (**a**) A representative melt curve of standards (black line; 0 to 100% methylation) and samples of TAC (green) and MCC (blue) are shown. (**b**) Comparison of methylation status between TAC (green bar) and MCC (blue bar). Representative results from one of three independent experiments are shown as the mean ± SD and scatter plots with the full data set of technical replicates (n = 5 measurements per sample, n = 4 animals, with TAC and MCC samples obtained from the same animals). * *p* < 0.05 between the samples.

To further compare the methylation status of the Nrf2 promoter region, HRM analyses for the extent of methylation in the Nrf2 promoter region in TAC and MCC samples were performed (Figure 4). TAC exhibited a relatively low methylation level as compared to that in MCC (Figure 4a). Quantification clearly demonstrated the statistically significant higher rate of methylation in MCC as compared to that in TAC (Figure 4b). These findings are consistent with our pyrosequencing analysis of CpG methylation in the Nrf2 promoter region, which similarly indicated elevated methylation levels in MCC samples.

## 4. Discussion

**Figure 5:**
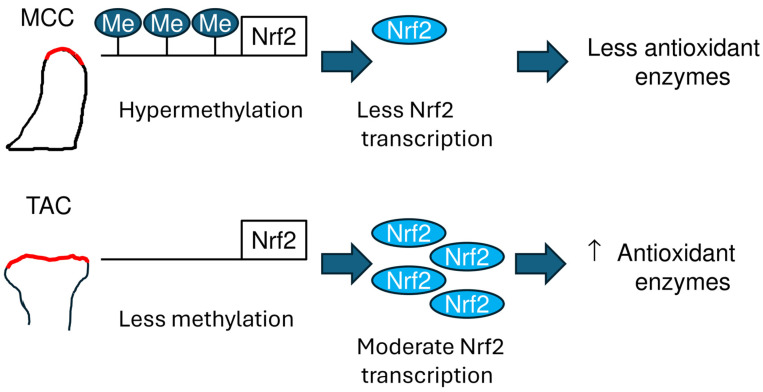
Schematic illustration of our proposed hypothesis. Higher methylation of the Nrf2 promoter in MCC is associated with reduced Nrf2 transcription and decreased antioxidant enzyme expression compared to TAC. This epigenetic–antioxidant relationship may compromise the antioxidant capacity of MCC, potentially explaining its heightened vulnerability to mechanical stress-induced degeneration.

In the present study, we discovered that the Nrf2 promoter region shows higher methylation levels in MCC compared to TAC, which is associated with low expression of Nrf2 and Nrf2-regulated antioxidant enzymes. This association may contribute to the low antioxidant capacity of MCC, as evidenced by increased 8-OHdG immunoreactivity indicating elevated oxidative DNA damage. As cartilage tissue is vulnerable to oxidative stress due to its avascular nature and hypoxic environment [8], this reduced antioxidant capacity likely underlies the lower resistance to mechanical stress in MCC. Figure 5 summarizes our proposed hypothesis: higher methylation of the Nrf2 promoter in MCC is associated with reduced Nrf2 transcription and decreased antioxidant enzyme expression, which may compromise the antioxidant capacity and potentially explain MCC’s heightened vulnerability to mechanical stress-induced degeneration.

Our results suggest that the higher methylation status of the Nrf2 promoter region is associated with reduced mechanical stress resistance, though several limitations should be noted. First, and most importantly, we did not conduct rescue experiments through demethylation of the Nrf2 promoter region to establish a causal relationship between promoter methylation and Nrf2 expression. We did not employ conventional genome-wide demethylating agents (e.g., 5-aza-2′-deoxycytidine) for this purpose, because their non-specific action across the genome would preclude attribution of any resulting change in Nrf2 expression specifically to demethylation of the Nrf2 promoter rather than to off-target epigenetic effects elsewhere in the genome. Establishing this locus-specific causal link will instead require targeted epigenome-editing approaches, such as CRISPR–dCas9 fused to the catalytic domain of TET1 (dCas9-TET1), directed to the CpG sites identified in the present study (Figure 1); we have identified this as a priority for future work. Therefore, while our data demonstrated a strong correlation, the direct mechanistic link requires further validation through functional experiments such as demethylation studies or site-specific mutagenesis. Second, we observed discrepancies in methylation values between pyrosequencing and HRM analysis, likely because HRM excels at detecting relative differences but may yield different absolute values [23]. Third, the relatively modest sample size (n = 4–8 biological replicates per analysis) may limit generalizability, although technical triplicates and consistent results across multiple independent experiments with sufficient statistical power support the validity of our findings. Fourth, although nucleic acid quality was monitored by spectrophotometric A260/280 ratio for genomic DNA and by microcapillary electrophoresis for RNA (Supplementary Figure S1), a dedicated capillary electrophoresis-based integrity assay for genomic DNA was not performed on these LCM-derived samples, owing to the limited tissue volume obtained by microdissection. We note, however, that the successful, concordant results obtained from two independent methylation assays (pyrosequencing and HRM) and two independent expression assays (RT-PCR and microarray) support that the extracted nucleic acids were of adequate quality for the analyses performed. Fifth, the present study was designed to compare MCC and TAC under their native, physiologically loaded states, which preserves each tissue’s authentic, long-term mechanical loading history but does not, by itself, establish whether mechanical loading is causally required to establish or maintain the epigenetic differences observed; this question is addressed further below in the context of future directions.

The differential mechanical stress resistance between MCC and TAC appears to be linked to their distinct developmental origins and matrix compositions. MCC derives from neural crest cells while articular cartilage originates from mesoderm [2]. Furthermore, MCC is enriched in type II collagen and proteoglycans, whereas articular cartilage contains higher amounts of type I collagen [3]. These compositional differences may affect mechanical properties and stress distribution. Our findings align with clinical observations demonstrating MCC growth inhibition under long-term compressive loading [4,5], while articular cartilage maintains resistance to compressive forces [6].

Despite MCC’s higher type II collagen content and greater chondrocyte proliferative capacity [7], our findings suggest that epigenetically suppressed antioxidant defenses significantly impair its capacity to manage oxidative stress from mechanical loading. Given that oxidative stress is a major factor in cartilage degeneration and osteoarthritis [9], the compromised antioxidant capacity in MCC, evidenced by elevated 8-OHdG levels, directly impacts tissue integrity under mechanical stress. This molecular mechanism explains why MCC exhibits paradoxically lower resistance to mechanical loading despite its specialized structure.

Our analyses revealed striking Nrf2 expression differences between MCC and TAC. MCC exhibited approximately 1/10 the Nrf2 expression of TAC at mRNA level and only 5% at protein level, while Keap1 expression remained constant, resulting in a dramatically lower Nrf2/Keap1 ratio in MCC. This imbalance likely diminishes Nrf2 signaling efficiency, as Keap1 is the principal negative regulator of Nrf2 [12]. It should be noted, however, that Keap1 was assessed only at the mRNA level in this study; because Keap1 regulates Nrf2 primarily through Cullin3-dependent ubiquitination and proteasomal degradation at the protein level, an unchanged Keap1 mRNA level does not exclude the possibility of altered Keap1 protein abundance or activity through post-transcriptional or post-translational mechanisms. Direct assessment of Keap1 protein level, for example by Western blot or immunohistochemistry, will be necessary in future work to fully evaluate the contribution of Keap1-mediated Nrf2 degradation to the phenotype observed here. Consequently, MCC showed significantly decreased expression of downstream antioxidant enzymes (NQO1, G6PD, HO-1), which constitute a critical defense against reactive oxygen species [14]. The elevated 8-OHdG levels in MCC provide compelling evidence of greater oxidative DNA damage due to insufficient antioxidant enzyme expression [21]. Recent studies support that Nrf2 pathway activation alleviates temporomandibular joint degeneration caused by overloading [15], while Nrf2 inhibition results in loss of protective effects [14].

Mechanical loading generates ROS through multiple mechanisms [24]. In tissues with robust Nrf2 signaling like TAC, ROS are effectively neutralized by antioxidant enzymes. However, in MCC where Nrf2 expression is lower and correlates with increased promoter methylation, the reduced antioxidant response may allow ROS accumulation and oxidative damage [25]. Furthermore, Nrf2 regulates inflammation and matrix metabolism in cartilage [26], suppressing pro-inflammatory cytokines [27] and matrix-degrading enzymes while promoting matrix component synthesis [28]. Therefore, reduced Nrf2 expression in MCC may impair matrix integrity maintenance under mechanical stress, further contributing to lower mechanical stress tolerance.

The present study demonstrates significant differences in epigenetic regulation of Nrf2 between MCC and TAC. The Nrf2 promoter region in MCC exhibits higher DNA methylation levels, as confirmed by HRM analysis. DNA methylation is a well-established epigenetic gene silencing mechanism [17], and hypermethylation of the Nrf2 promoter suppresses its expression in cancer cells [16]. Based on these established mechanisms in other tissues, our results suggest that a similar epigenetic regulatory mechanism may be associated with reduced Nrf2 expression and diminished antioxidant capacity in MCC, though direct functional validation is required.

While our study focused on DNA methylation, other epigenetic mechanisms may also contribute to differential Nrf2 expression. Histone modifications, particularly H3 acetylation, positively regulate Nrf2 expression [16], while microRNAs such as miR-28 can suppress Nrf2 post-transcriptionally [29]. The interplay between these mechanisms likely creates a complex regulatory network determining tissue-specific Nrf2 expression patterns. Rescue experiments such as Nrf2 promoter demethylation are necessary to clarify the mechanism.

Our findings provide novel insights into epigenetic mechanisms underlying differential mechanical stress resistance between MCC and TAC. We demonstrated that Nrf2 promoter hypermethylation in MCC reduces Nrf2 expression, subsequently diminishing antioxidant capacity and increasing susceptibility to oxidative damage under mechanical stress. These findings have significant implications for understanding temporomandibular joint disorder pathogenesis. While mechanical overloading is a primary risk factor for temporomandibular joint osteoarthritis [30], the molecular mechanisms remain unclear. Our discovery of epigenetic Nrf2 pathway regulation establishes a direct link between epigenetic suppression of antioxidant capacity and mechanical stress vulnerability.

The clinical relevance extends beyond temporomandibular disorders. Recent studies demonstrated that Nrf2 signaling activation protects against cartilage degradation in osteoarthritis by reducing oxidative stress and inflammation [26,31]. More broadly, DNA methylation has emerged as a key regulatory mechanism in osteoarthritic cartilage degeneration [18]. By identifying Nrf2 promoter hypermethylation as a determinant of antioxidant capacity specifically in MCC, our findings suggest that an analogous epigenetic–oxidative stress axis may operate in mechanically loaded cartilage more broadly, including weight-bearing joints affected by osteoarthritis, and merit investigation in that context. Nrf2 activation has additionally been reported to inhibit ectopic calcification by transcriptionally upregulating ENPP1 [32], further supporting Nrf2 as a multifunctional therapeutic target relevant to cartilage and joint pathology. Our study suggests epigenetic interventions targeting Nrf2 promoter DNA methylation could represent a novel therapeutic approach. Future research should explore demethylating agents or HDAC inhibitors in restoring Nrf2 expression and enhancing mechanical stress resistance in MCC. In parallel, since the present study compared MCC and TAC under their native physiological loading conditions, future work incorporating in vivo mechanical overloading models (e.g., excessive occlusal loading or surgically induced condylar overloading) or in vitro cyclic/compressive loading of primary MCC and TAC chondrocytes will be needed to determine whether superphysiological mechanical stress dynamically modulates Nrf2 promoter methylation and antioxidant capacity, thereby more directly linking mechanical overloading to the epigenetic phenotype described here. Additionally, investigating upstream regulators of cartilage-specific epigenetic patterns and age-related epigenetic changes affecting cartilage antioxidant capacity would be valuable, as temporomandibular disorders and osteoarthritis show age-dependent prevalence [33,34]. Given the reversible nature of epigenetic modifications, targeting the Nrf2–antioxidant pathway offers promising avenues for developing preventive and therapeutic strategies for cartilage disorders.

## 5. Conclusions

In conclusion, our study demonstrates that higher Nrf2 promoter methylation in mandibular condylar cartilage is associated with reduced Nrf2 expression and diminished antioxidant capacity, potentially contributing to its heightened vulnerability to mechanical stress. While further functional studies are needed to establish causality, these findings suggest that epigenetic regulation of the Nrf2 pathway may represent a novel therapeutic target for temporomandibular joint disorders and related cartilage pathologies.

An important open question raised by these findings is why Nrf2 promoter methylation is so markedly elevated specifically in MCC. Because MCC shares its secondary-cartilage developmental origin with other craniofacial secondary cartilages, including the mandibular angular and coronoid process cartilages [35], and the secondary cartilage of the clavicle [36], future studies should determine whether this Nrf2 epigenetic axis is a general feature of secondary cartilage as a class or specific to the condyle, and whether comparable promoter hypermethylation contributes to age-related degeneration of primary articular cartilage more broadly.

## Figures and Tables

**Table 1:** Microarray analysis for gene expressions of antioxidation-related genes.

Gene Name	Signal Intensity		Expression Ratio (MCC/TAC)
	MCC	TAC	
Nrf2	810.8	8657.7	0.094
Keap1	2412.6	2412.8	1
Nrf2/Keap1 ratio	0.336	3.588	0.094
Heme oxygenase-1 (HO-1)	495.4	1106.7	0.448
NAD (P) H quinone oxidoreductase 1 (NQO1)	268.6	5180.5	0.052
NAD (P) H quinone oxidoreductase 2 (NQO2)	2526.6	6053.6	0.417
Glutamate-cysteine ligase, catalytic subunit (GCS)	181.2	3405	0.053
Microsomal glutathione S-transferase 1	18.1	6691.8	0.003
Glutathione S-transferase kappa 1	5970	13,314.3	0.448
Glucose-6-phosphate dehydrogenase X-linked (G6PD)	5037.7	11,940.4	0.422
Peroxiredoxin 3	2793.1	13,491.1	0.207
Peroxiredoxin 4	21	110.5	0.19
Peroxiredoxin 6	47.7	306.8	0.156

**Table 2:** Percent of methylation in CpG measured by pyrosequencing.

Sample	Site-1	Site-2	Site-3	Site-4	Site-5	Mean ± SD
MCC	21.57	28.31	12.9	5.11	9.71	15.52 ± 9.34
TAC	18.66	14.04	2.36	0	4.32	7.88 ± 8.05
Difference (MCC-TAC)	2.91	14.27	10.54	5.11	5.39	7.64 ± 4.64

## Data Availability

Data is available in a publicly accessible repository (https://doi.org/10.6084/m9.figshare.32323776). Other data sets used and/or analyzed during the current study are available from the corresponding author upon reasonable request.

## Supplementary Materials

The following are available online at https://www.mdpi.com/article/10.3390/antiox15070854/s1, Figure S1: Quality control of laser-microdissected cartilage and extracted RNA. (A, B) Representative photomicrographs showing the boundary of the laser-microdissected (LMD) region for mandibular condylar cartilage (MCC, A) and tibial articular cartilage (TAC, B). Scale bar: 300 mm (A), 150 mm (B). (C, D) Representative Bioanalyzer electropherograms showing RNA integrity for MCC (C) and TAC (D). Two clearly resolved peaks corresponding to 18S and 28S ribosomal RNA are observed in both samples, with minimal degradation products, confirming that RNA of sufficient quality was obtained from the microdissected tissue for downstream gene expression analysis (real-time RT-PCR and microarray).
