# Supplementary material for: Promoter Hypermethylation Is Associated with Reduced Nrf2 and Antioxidant Enzyme Expression in Mandibular Condylar Cartilage in Mice
Source: Antioxidants (Basel). 2026 Jul 6;15(7):854. doi: 10.3390/antiox15070854 (PMC13403435; doi:10.3390/antiox15070854)
Supplement: Supplementary file 1 [file antioxidants-15-00854-s001.zip › 20260622Figure legend for supplementary figure.pdf]

Figure legend for supplementary figure.

Supplementary Figure S1. Quality control of laser-microdissected cartilage and extracted RNA.

(A, B) Representative photomicrographs showing the boundary of the laser-microdissected (LMD) region for mandibular condylar cartilage (MCC, A) and tibial articular cartilage (TAC, B). Scale bar: 300 μm (A), 150 μm (B).

(C, D) Representative Bioanalyzer electropherograms showing RNA integrity for MCC (C) and TAC (D). Two clearly resolved peaks corresponding to 18S and 28S ribosomal RNA are observed in both samples, with minimal degradation products, confirming that RNA of sufficient quality was obtained from the microdissected tissue for downstream gene expression analysis (real-time RT-PCR and microarray).
