# Supplementary figures and images for: Promoter Hypermethylation Is Associated with Reduced Nrf2 and Antioxidant Enzyme Expression in Mandibular Condylar Cartilage in Mice
Source: Antioxidants (Basel). 2026 Jul 6;15(7):854. doi: 10.3390/antiox15070854 (PMC13403435; doi:10.3390/antiox15070854)

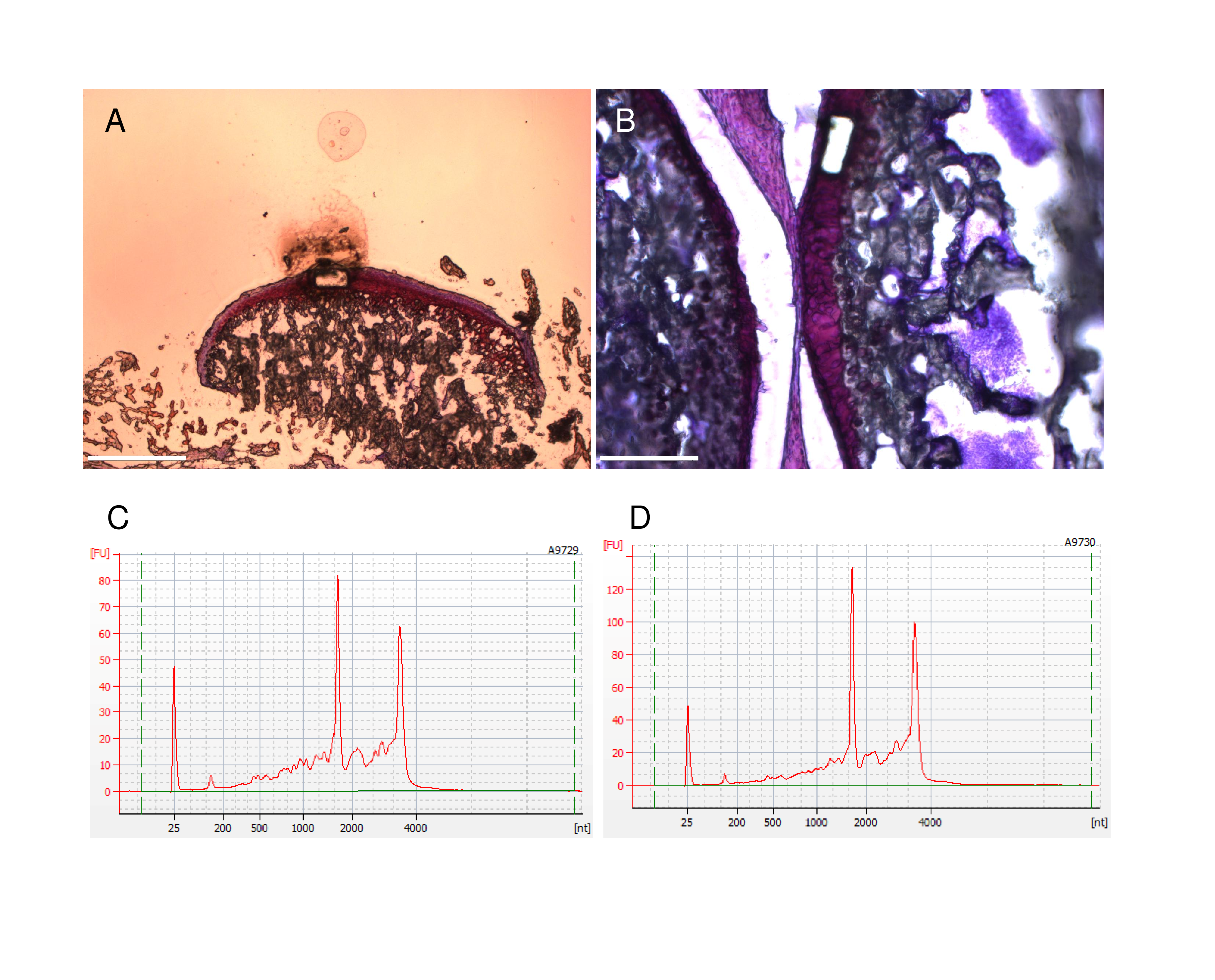

Supplement: Supplementary file 1 [file antioxidants-15-00854-s001.zip › Figure S1.tif]
